# The COVID-19 Pandemic: Clinical Information for Ophthalmologists

**DOI:** 10.4274/tjo.galenos.2020.29805

**Published:** 2020-04-29

**Authors:** Banu Bozkurt, Sait Eğrilmez, Tomris Şengör, Özlem Yıldırım, Murat İrkeç

**Affiliations:** 1 Selçuk University Faculty of Medicine, Department of Ophthalmology, Konya, Turkey; 2 Ege University Faculty of Medicine, Department of Ophthalmology, İzmir, Turkey (E); 3 İstanbul Bilim University Faculty of Medicine, Department of Ophthalmology, İstanbul, Turkey (E); 4 Mersin University Faculty of Medicine, Department of Ophthalmology, Mersin, Turkey; 5 Hacettepe University Faculty of Medicine, Department of Ophthalmology, Ankara, Turkey (E)

## Introduction

The coronavirus epidemic that started in China has rapidly spread to all countries of the world and caused a significant number of deaths. The situation was first reported to the World Health Organization (WHO) China office on December 31, 2019 as an outbreak of pneumonia of unknown cause in the city of Wuhan (population ~11 million) in the Hubei province.^1,2,3,4^ The disease was believed to have originated in a seafood market, which was closed for disinfection on January 1, 2020. Of 44 cases reported on January 3, 2020, 11 patients had severe disease while 33 patients were stable.^2,3^ On January 7, 2020, it was determined that the epidemic was caused by a novel coronavirus (nCoV). Thailand reported its first case on January 13 and Japan on January 15, while on January 20 the first case was reported in Korea and 6 were reported dead in the city of Wuhan. Later, it appeared in countries such as the United States, Vietnam, Singapore, and Australia, and spread to the European nations, starting with France on January 25, 2020. The WHO reported that the first cases in Wuhan had been infected via animals, after which the virus was transmitted from person to person and detected in clusters among families.^3^ On January 30, 2020, the epidemic was recognized as a public health emergency of international concern, and on February 11, 2020, the WHO named the novel coronavirus disease COVID-19. The International Committee on Taxonomy of Viruses named the new virus SARS-CoV-2 (severe acute respiratory syndrome coronavirus 2) and the WHO declared the coronavirus epidemic a pandemic on March 12.^4^ On April 2, the WHO website reported 896,450 infected individuals worldwide, 206 affected countries, and 45,526 deaths globally.^5^

In Turkey, a Coronavirus Scientific Committee including academicians working in university departments such as infectious diseases, intensive care, pulmonology, emergency medicine, and public health was formed under the Ministry of Health on January 10, shortly after WHO announced the epidemic.^6^ Thermal cameras were installed in Turkish airports and additional scanning was implemented, especially for passengers arriving from China. As the epidemic spread to other countries, screening was expanded to include passengers from countries with reported cases, and any individuals showing signs of coronavirus infection were quarantined. In February, flights to all countries with growing outbreaks were suspended. In the first week of March, hand disinfectant stations were placed in mass transit and public areas in some provinces. The first case of COVID-19 in Turkey was announced by the Ministry of Health on March 10, 2020. Schools were closed on March 13 and some other precautions were implemented, such as not allowing spectators at sporting events and requiring special permission for government personnel to leave the country. The first COVID-related death in Turkey occurred on March 15, 2020. As of March 19, all sports, scientific, cultural, and artistic activities have been postponed. Lockdown measures were later introduced, first for individuals over the age of 65, then for those under the age of 20. On April 1, it was reported that 601 healthcare workers were infected and 1 physician had died.^6^ As of April 2, there were 18,135 infected individuals and 356 deaths in Turkey.

## The Causative Pathogen

Coronaviruses are enveloped, single-chain RNA viruses, and 4 types of human coronaviruses (HCoV-229E, -NL63, -OC43, and -HKU1) have been reported to cause upper respiratory infections and colds. Coronaviruses of animal origin (SARS-CoV 2002, MERS-CoV 2012, 2019-nCoV/SARS-CoV-2) can lead to fatal respiratory failure in humans.^7,8,9^ Coronaviruses bind to respiratory epithelial cells and enteric cells, causing cytopathic changes.

In this pandemic, bronchoalveolar lavage samples from a COVID-19 patient first tested positive for pan-betacoronavirus with real-time PCR (RT-PCR).^3^ Whole-genome sequencing of the virus was done by Illumina and nanopore sequencing, and bioinformatic analysis revealed that the virus carries the typical features of the coronavirus family and phylogenetically belongs to the 2B lineage of betacoronaviruses. When the COVID-19 virus and other betacoronavirus genome sequences were compared, it was found that the novel coronavirus shows 96% similarity to the bat SARS-like coronavirus strain BatCovRaTG13 and that the spike (S) protein on the virus binds to angiotensin-converting enzyme 2 (ACE2) on the cell surface.^3,4,9,10,11^

## Clinical Symptoms

Although COVID-19 is largely asymptomatic or mild (80%), it can sometimes lead to severe pneumonia and death.^12,13^ It is generally more severe in individuals over 60 years of age and those with comorbid diseases such as hypertension, cardiovascular disease, chronic lung disease, and cancer. It is less common and milder in children. The incubation period is 1-14 days (mean 5-6 days) and the classic signs and symptoms reported include high fever, dry cough, shortness of breath, muscle pain, and fatigue, with bilateral ground-glass opacity on chest tomography.^12,13^ The presence of conjunctivitis has not been reported in these studies. An article published in the New England Journal of Medicine in late February evaluated the clinical symptoms and outcomes of 1,099 of the 7,736 patients who were hospitalized due to a diagnosis of COVID-19 in 552 hospitals in China through January 29, 2020.^14^ The median age of the patients was 47 years; 41.9% were female, and the incubation period was 4 days. Only 0.9% of patients were under the age of 15, while 23.7% had a comorbidity such as hypertension or chronic obstructive pulmonary disease. The most common symptom was fever (88.7%), followed by cough (67.8%). Nausea, vomiting, and diarrhea were rarely observed. Lymphocytopenia was present in 83.2% and ground-glass opacities were observed on lung tomography in 56.4% of patients. Five percent of the patients were admitted to intensive care units, 2.3% required mechanical ventilation, and 1.4% died. Conjunctival congestion was detected in 9 (0.8%) of the patients.^14^

## Diagnosis

COVID-19 is diagnosed based on detection of genetic material from the virus by molecular microbiological methods in a patient specimen. Nasopharyngeal or saliva samples obtained from suspected patients with high fever, travel history, and contact with infected patients, as well as the close contacts of these suspected patients, are tested using specific RT-PCR kits for 2019-nCoV to detect RdRp and the variable S gene.^15^ In addition, serum IgM and IgG are also analyzed to identify active or recovered cases.

## Transmission

Transmission of COVID-19 is known to mostly occur to individuals in close contact with symptomatic patients via airborne microdroplets, and through direct contact with infected individuals or contaminated objects.^16,17^ Social isolation and personal protection are extremely important for preventing spread. Virus-laden microdroplets released into the environment by sneezing, coughing, and exhaling can come into contact with the mouth, nasal mucosa, and conjunctiva. For this reason, the WHO states that healthcare workers who are in contact with a suspected COVID-19 patient must protect their eyes, mouth, and nose with goggles, masks, filtering masks (N95, FFP2, FFP3), and face shield.^16^

## SARS-CoV-2 and Ocular Involvement

Transmission of the novel coronavirus through ocular fluids is a cause of serious concern for ophthalmologists. On January 22, Guangfa Wang, a physician specializing in pneumonia, developed conjunctivitis while visiting Wuhan for inspection. He later tested positive for SARS-CoV-2 and suggested that ocular infection was an alternative route of transmission of the virus.^18^ Li Wenliang, an ophthalmologist working in Wuhan, contracted the disease in early January after contact with a glaucoma patient and later lost his life.^19^ A report published in The Lancet in February 2020 and an editorial published in the British Journal of Ophthalmology in March stated that, in light of previous publications regarding coronavirus and SARS, the ocular surface is a potential target tissue for SARS-CoV-2 invasion.^20,21^ Certain coronaviruses are known to cause conjunctivitis in humans.^22,23^ Of the human coronaviruses, NL 63 (HCoV-NL63) was first isolated in an infant with bronchiolitis and conjunctivitis,^22^ and a later publication reported that conjunctivitis was present in 17% (n=3) of 18 children with respiratory infection whose nasal swabs had tested positive for HCoV-NL63.^23^

Loon et al.^24^ published a study in Singapore in which they collected tear samples from 36 patients followed for 12 days for suspected SARS and analyzed them using PCR. Eight of these patients were later serologically diagnosed with SARS, while tear samples of 3 patients (37.5%) tested positive by PCR. Tear test results were negative in the remaining suspected cases. It was reported that in all patients with positive tear PCR results, the samples had been collected at an early stage. The authors stated that collecting a tear sample is extremely simple and reproducible and that it can therefore be used for diagnostic purposes in the early stage. They noted that ophthalmologists and other healthcare professionals work within close proximity to patients’ eyes and that this may be a source of infection, citing Goldmann applanation tonometry, contact lens fitting, and spectacle frames as potential transmitters of infection. They also stated that for this reason, healthcare workers’ compliance with personal protective equipment guidelines (M3G: mask, gown, gloves, and goggles/face shield) is imperative during the examination and treatment of SARS patients.^24^ The debate still continues regarding how the SARS-CoV is found in tears.^9^ Mechanisms discussed include transmission through droplets, upstream passage from an upper respiratory tract infection through the nasolacrimal duct, and from hematogenous infection of the lacrimal gland.

In a study published by Chan et al.^25^ in the same year (2004), nasopharyngeal, stool, tear, and conjunctival swab samples were collected from 20 SARS patients, 17 of whom were confirmed cases. The nasopharyngeal swab and stool samples of 5 (29.4%) of the 17 patients tested positive for SARS-CoV when tested by PCR, while SARS-CoV could not be detected with RT-PCR or viral culturing in any of the tear/conjunctival swabs. Several possible explanations for the negative test results were suggested. The results may have been false-negatives, and collecting more samples could improve sensitivity, or the virus and its genetic material may be detectable in tears only in a short window during the disease, or the virus is not present in tears. The authors concluded that checking for the virus in tears or conjunctival swabs had no place in disease screening.^24^

Being a relatively new entity, there are extremely few studies on COVID-19. In a study conducted in China, Xia et al.^26^ collected tears, conjunctival swab, and saliva samples twice from 30 COVID-19 patients. Only the 2 samples from a patient with conjunctivitis tested positive in RT-PCR, while the other 58 tear samples tested negative. The patient with conjunctivitis was reported to have conjunctival congestion and serous discharge; however, the virus could not be isolated. Of the 60 saliva samples, 55 yielded positive results. The authors stated that even though the chance of virus being present in tear and conjunctiva samples is low, this does not mean that the conjunctiva cannot act as a portal of entry for the virus. Because ophthalmologists are within close range of patients during examination, the patient’s saliva may splash on the face and cause infection, making the use of safety goggles an absolute necessity.

Finally, according to an article by Jun et al.^27^ published in the journal *Ophthalmology* last week, viral culture and RT-PCR analysis of 64 tear samples collected simultaneously with nasopharyngeal swabs from 17 COVID-19 patients between 3 and 20 days after initial symptom onset failed to demonstrate the presence of 2019-CoV. Ocular symptoms were not observed in any of the patients, but 1 patient developed conjunctival redness and chemosis while in hospital. Although these results may seem comforting, they have led to arguments that the negative results could be attributed to the absence of active conjunctivitis at time of sample collection, the small number of conjunctiva and tear samples, and the fact that the samples were collected 2 to 3 weeks after symptom onset, when there is lower virus load.^28^

According to another argument, as SARS-CoV-2 enters cells by binding with S proteins to ACE2 in the respiratory and pulmonary epithelia, and because ACE2 is not expressed in the conjunctival or corneal epithelium^29^ but is expressed only in the retinal and retinal pigment epithelium, this virus may enter the tears through droplets and then be transferred to the respiratory tract through the nasolacrimal canal, and the use of safety goggles by healthcare professionals is therefore recommended.^30^

## Considerations for Ophthalmologists During the COVID-19 Pandemic

Seitzman and Doan^28^ stated that the healthcare industry accounts for 11% of professional groups in the US and that occupational exposure to the virus occurs mostly by transmission through infected airborne droplets. They noted that the risk of exposure to this infection is much higher during slit-lamp examination and other ophthalmologic imaging measurements where there is closer face-to-face contact, because the virus load is especially high in the nasal cavity. As SARS-CoV-2 can survive in air for at least 3 hours,^31^ they recommend not talking during slit-lamp examination and keeping the examination as brief as possible.

In the American Academy of Ophthalmology (AAO) guidelines^32^ and a review by Lai et al.^19^ sharing their experiences regarding infection control in ophthalmology practice during the COVID-19 pandemic, it is recommended that patient examination only be conducted in emergency circumstances and that patients always be screened for SARS-CoV-2 prior to ophthalmic examination (FTOCC: fever or symptoms of respiratory tract infection; recent travel history; occupation [healthcare professional], contact with an individual who has COVID-19, and presence of certain symptoms in the family [cluster]). It is also advised to postpone appointments at least 14 days for individuals suspected of having COVID-19 and to regard patients with conjunctivitis as contagious carriers.

The following guidelines were specified for performing ophthalmic examination in emergencies:^19,28,32^

• Patient numbers should be reduced; patients should be informed by message or phone call not to come to the office except for emergencies.

• Prescriptions and reports should be approved without an office visit.

• For patients who absolutely must come in, examination times should be defined within a schedule.

• Care should be taken to maintain appropriate social distance in examination rooms.

• Examination of patients with suspected COVID-19 should be conducted in a separate, dedicated room.

• Examination devices and any other surfaces in the room touched by hand should be cleaned with 0.1% sodium hypochlorite or 70% ethanol for at least 1 minute before and after a patient examination. Commercially available bleach contains 5% sodium hypochlorite.

• Patients should wear a face mask.

• Hands should be washed frequently with soap and water.

• Ophthalmologists should always wear an N95 mask and safety goggles or face shield during examination and gloves should be changed between patients.

• Healthcare workers should be checked for fever and any symptoms should be reported immediately.

• Visual acuity should be assessed from a distance.

• Shields acting as a barrier between patient and physician should be placed on biomicroscopes and disinfected between exams.

• Clinical examination should be diagnostic and brief.

• Air-puff tonometers should not be used because they are a potential source of microaerosols. Intraocular pressure should be measured using tonometers with disposable tips. If Goldmann tonometry is used, tips should be disinfected before and after each patient.^33^

• All non-urgent elective surgeries, procedures (e.g., contact lens fitting), and testing (e.g., electrodiagnostic testing) should be postponed to a later date.

• Imaging should be done only if essential for diagnosis and will influence treatment.

• When necessary, emergency surgery should be performed under local anesthesia if possible, and a COVID-19 test should be performed if the patient has fever or other suspicious symptoms.

• It should be remembered during this period that conjunctivitis can also be caused by viruses other than 2019-nCoV.

## Is Eye Examination Necessary When Using Chloroquine/Hydroxychloroquine?

Chloroquine and hydroxychloroquine are drugs shown to be effective against the SARS virus, and approximately 10 clinical trials are underway in the current pandemic.^34,35^ In China, patients are treated with 500 mg of chloroquine twice daily or 400 mg of hydroxychloroquine 4 times a day for 10 days. Chloroquine and hydroxychloroquine are well-known drugs among ophthalmologists as they cause retinal toxicity with long-term use for the treatment of rheumatoid diseases. The AAO guidelines state that the risk of developing retinopathy within 10 years is extremely low if used at doses less than 5 mg/kg/day.^36^ In a study including 22 patients in France, COVID-19 positive patients were given 600 mg of hydroxychloroquine per day for 10 days and it was observed that virus load decreased by 50% when used alone and by up to 100% when used in combination with azithromycin.^37^ Although the dose cited by the Chinese group is much higher than the dose used in rheumatoid patients, they reported that short-term use (less than 2 weeks) will not cause any toxicity and that conducting detailed eye exams before and after this treatment during the pandemic is unnecessary.^34^

The Turkish Ministry of Health, in a guidance report entitled “Evaluation of Healthcare Workers with Patient Contact” published on March 25, 2020, identified ophthalmologic examination as a procedure requiring intensive contact, and recommended prophylaxis with hydroxychloroquine for a total of 3 days (400 mg twice on day 1, 200 mg twice daily on days 2 and 3) and 5 days of home isolation followed by a PCR test in the event of high-risk contact with COVID-19 patients without the use of personal protective equipment.^38^ The Turkish Society of Clinical Microbiology and Infectious Diseases reported, based on available data, that these agents should only be used by some symptomatic COVID-19 patients, that they should be initiated early, and should not be used for prophylaxis.^39^ Treatment algorithms from the Ministry of Health recommend hydroxychloroquine for adults with uncomplicated probable/confirmed COVID-19, with oseltamivir if influenza is suspected; hydroxychloroquine and azithromycin for adults with indication for hospitalization; and hydroxychloroquine, azithromycin, and favipiravir for adults with COVID pneumonia.^40^

Treatment with lopinavir/ritonavir is recommended for pregnant women. For children, the first-line treatment options are oseltamivir, hydroxychloroquine, and azithromycin.^41^ In case of progression, lopinavir/ritonavir therapy can be initiated.

In summary, close-proximity examination procedures and those requiring physical contact pose a high risk for the transmission of SARS-CoV-2 to ophthalmologists. Therefore, examination procedures such as ophthalmoscopy, biomicroscopy, and manifest refraction should not be performed without personal protective equipment. Disinfection of devices and instruments should be made a routine part of examination for all procedures that require contact with the ocular surface. All surfaces touched both inside and outside the office by patients during their visits, including the examination room and outside surfaces such as the handle of the entrance door, doorbell, and elevator buttons, require regular disinfection. Therefore, the number of patients should be reduced except for urgent cases that cannot be postponed. It is extremely important to educate patients in order to prevent infection via the ocular surface. Patients should be advised to stop rubbing their eyes habitually and to avoid all hand contact with the eyes before washing appropriately. Hygiene rules should be adhered to strictly, especially when using contact lenses, and the use of eyeglasses should be recommended instead if necessary.
